# *ALK* variants, PD-L1 expression, and their association with outcomes in *ALK*-positive NSCLC patients

**DOI:** 10.1038/s41598-020-78152-1

**Published:** 2020-12-03

**Authors:** Gee-Chen Chang, Tsung-Ying Yang, Kun-Chieh Chen, Kuo-Hsuan Hsu, Yen-Hsiang Huang, Kang-Yi Su, Sung-Liang Yu, Jeng-Sen Tseng

**Affiliations:** 1 Division of Chest Medicine, Department of Internal Medicine, Taichung Veterans General Hospital, No. 1650, Sect. 4, Taiwan Blvd., Taichung, 407 Taiwan; 2 Faculty of Medicine, School of Medicine, National Yang-Ming University, Taipei, 112 Taiwan; 3 Institute of Biomedical Sciences, National Chung Hsing University, Taichung, 402 Taiwan; 4 Division of Pulmonary Medicine, Department of Internal Medicine, Chung Shan Medical University Hospital, Taichung, 402 Taiwan; 5 School of Medicine, Chung Shan Medical University, Taichung, 402 Taiwan; 6 Institute of Medicine, Chung Shan Medical University, Taichung, 402 Taiwan; 7 Department of Applied Chemistry, National Chi Nan University, Nantou, 545 Taiwan; 8 Division of Critical Care and Respiratory Therapy, Department of Internal Medicine, Taichung Veterans General Hospital, Taichung, 407 Taiwan; 9 Department of Clinical Laboratory Sciences and Medical Biotechnology, College of Medicine, National Taiwan University, Taipei, 100 Taiwan; 10 Department of Laboratory Medicine, National Taiwan University Hospital, Taipei, 100 Taiwan; 11 Center of Genomic Medicine, National Taiwan University College of Medicine, Taipei, 100 Taiwan; 12 Department of Pathology and Graduate Institute of Pathology, College of Medicine, National Taiwan University, Taipei, 100 Taiwan; 13 Center for Optoelectronic Biomedicine, College of Medicine, National Taiwan University, Taipei, 100 Taiwan

**Keywords:** Lung cancer, Cancer, Molecular biology

## Abstract

It remains unclear how programmed death-ligand 1 (PD-L1) expression interacts with *anaplastic lymphoma kinase* (*ALK*) mutation, its variants, and the outcome of treatment. One hundred and twenty four out of 1255 patients (9.9%) were deemed *ALK*-positive by the Ventana IHC assay. PD-L1 status and *ALK* variants were available in 100 and 59 patients, respectively. PD-L1 positive (TPS ≥ 1%) and strong positive (TPS ≥ 50%) rate was 50% and 16%, respectively. A total of 64 variant types were detected in 59 patients. V1 (32.8%) and V3a/b (28.1%) were the most common variants. There was no significant association between *ALK* variants and the PD-L1 expression. The presence of V3a/b subtype independently predicted a worse overall survival in patients receiving ALK inhibitor(s) (aHR 5.10 [95% CI 1.22–21.25], *P* = 0.025) and platinum plus pemetrexed (aHR 9.62 [95% CI 1.90–48.80], *P* = 0.006). While incorporating *ALK* variants and PD-L1 expression together, patients with non-V3a/b/positive PD-L1 showed a trend towards longer OS. In conclusion, *ALK*-positive NSCLC patients possess a high PD-L1 expression rate. Although there was no significant association between PD-L1 expression and *ALK* variants, the outcome of *ALK*-positive patients could be sorted by these two biomarkers.

## Introduction

In 2007, Soda et al. and Rikova et al. identified a fusion gene in lung cancer cells containing an inversion of the *echinoderm microtubule-associated protein-like 4* (*EML4*) with the *anaplastic lymphoma kinase* (*ALK*) that possesses oncogenic activity and can serve as a therapeutic target^1,2^. The mutation can be detected in approximately 3–5% of non-small cell lung cancer (NSCLC) patients with distinct clinicopathological features^3^. Several ALK inhibitors can effectively suppress the oncogenic activity of *ALK* rearrangement and provide a better outcome; hence, they have emerged as important front-line therapies in advanced *ALK*-positive lung cancer patients^4–8^.

*ALK* testing is recommended in advanced NSCLC patients, particularly those with adenocarcinoma histology. Although, fluorescent in situ hybridization, immunohistochemistry (IHC), and polymerase chain reaction (PCR)-based next-generation sequencing (NGS) are widely available methods for *ALK* testing, only the targeted NGS can recognize the *ALK* variants^9^. Many *ALK* variants have been reported according to the different fusion partners. However, the results of the distribution of these subtypes and their impact on patients’ outcome were not consistent^10–12^.

The interaction between driver mutations and immunological status, such as programmed death-ligand 1 (PD-L1) expression, may be diverse. In the case of *epidermal growth factor receptor* (*EGFR*) mutation, *EGFR*-mutant NSCLC patients have displayed a lower chance of PD-L1 expression and a strong PD-L1 expression has been reported to correlate with a worse outcome of EGFR-tyrosine kinase inhibitor (TKI) treatment^13–15^. Whether these phenomena also hold true in patients with *ALK* fusion remains unclear. Moreover, it is currently unknown whether there is an association between *ALK* variants and PD-L1 status and how it influences the efficacy of treatment. In the present study, we enrolled *ALK*-positive NSCLC patients to access their PD-L1 expression and *ALK* variants, analyze the relationship, and clarify their impacts on the outcome of treatment.

## Results

### ALK mutation in EGFR-wild type NSCLC

A total of 1255 *EGFR*-wild type NSCLC patients were enrolled for *ALK* mutation examination. The median age was 63 years, 530 patients (42.2%) were female, and 657 patients (52.4%) were non-smokers. Adenocarcinoma accounted for the major histological types (90.6%). Overall, 124 patients (9.9%) were *ALK*-positive.

The results of univariate analysis of *ALK* fusion and patients’ characteristics are summarized in Supplementary Table S2. Patients with an age ≤ 50 years, female gender, adenocarcinoma histology, and non-smokers were more likely to harbor *ALK* mutation (*P* < 0.001, = 0.005, 0.034, and 0.004, respectively). In multivariate analysis, an age ≤ 50 years (aOR 3.17 [95% CI 2.15–4.68], *P* < 0.001) and non-smokers (aOR 1.49 [95% CI 1.00–2.22], *P* = 0.048) both independently predicted the presence of *ALK* mutation.

**Figure 1:**
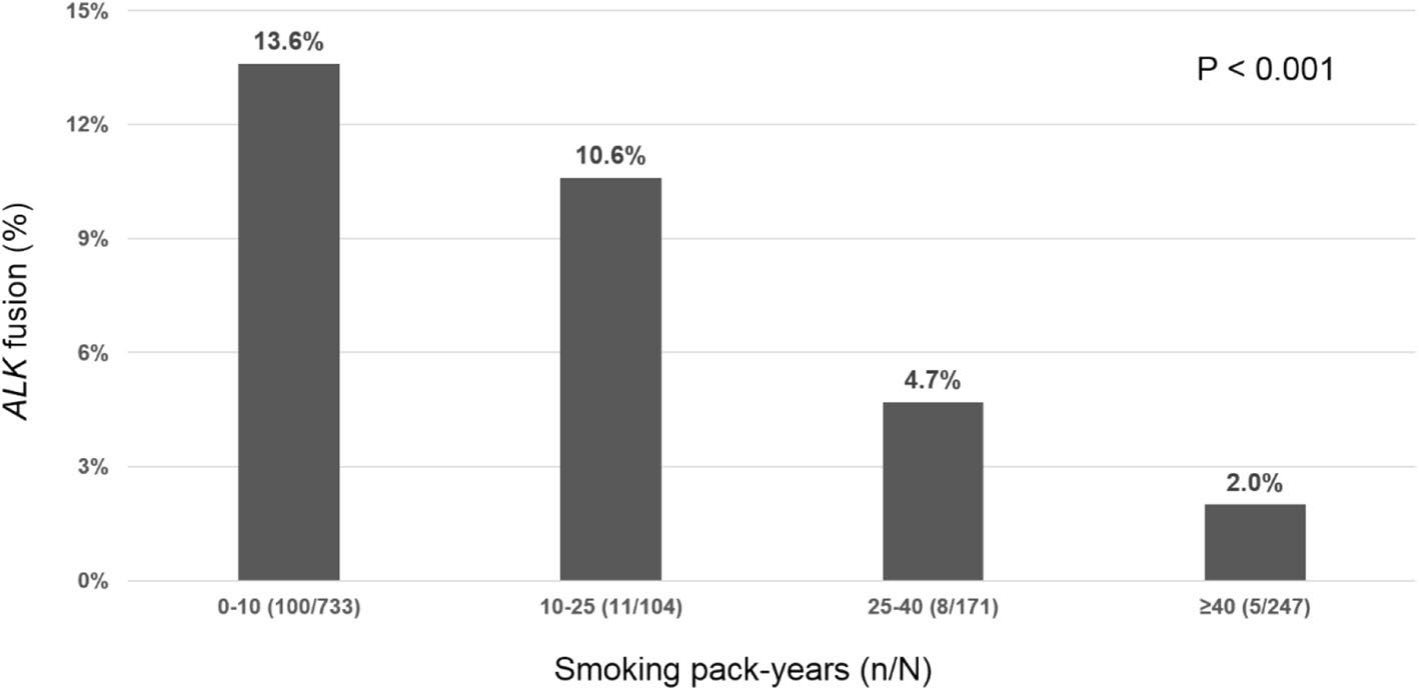
Association between cigarette smoking dose and *ALK* mutation (N, total cases; n, *ALK*-positive patients).

Figure 1 illustrates that the *ALK* mutation rate was reversely associated with the smoking pack-years (*P* < 0.001). Non-smokers and light smokers (≤ 10 pack-years) had the highest *ALK* mutation rate (13.6%).

### PD-L1 status and ALK variant subtypes

**Figure 2:**
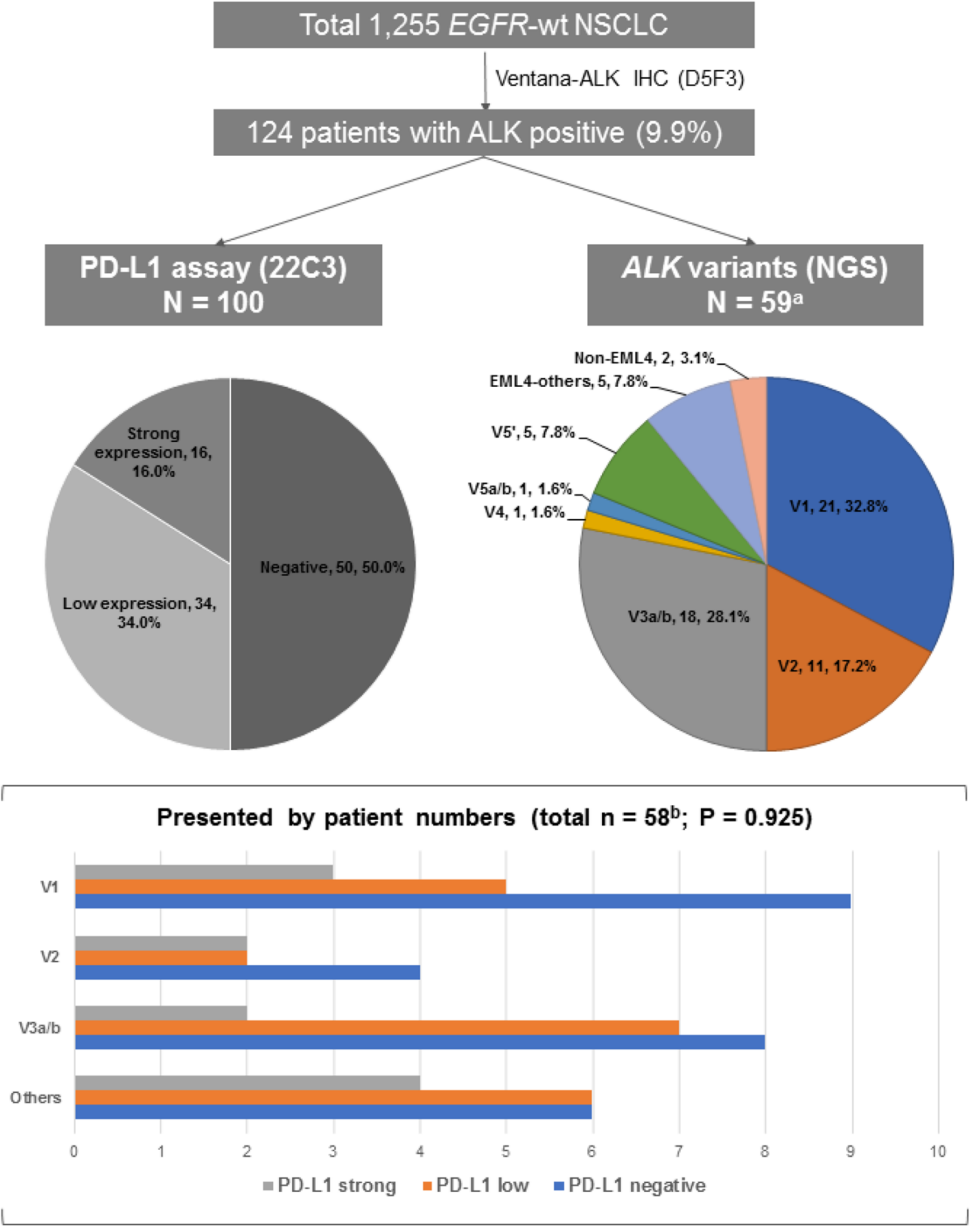
PD-L1 status and *ALK* variants in *ALK*-positive NSCLC patients (^a^Total 64 variants were detected in 59 patients; ^b^Patients with complex variants were categorized in the others).

Among the 115 patients prepared for PD-L1 assay, 15 specimens were considered as inadequate due to having less than 100 viable tumor cells. Among the 87 patients prepared for *ALK* variants analysis, 28 specimens did not meet the adequacy for NGS testing and most of them were cytological specimens. Finally, a total of 100 patients had PD-L1 expression results, while 59 patients had detectable *ALK* variants (Fig. 2).

Fifty patients (50.0%) were PD-L1 negative, 34 patients (34.0%) were PD-L1 low expression (tumor proportion score [TPS] 1–50%), and 16 patients (16.0%) had a strong PD-L1 expression (TPS ≥ 50%). There was no significant association between the PD-L1 expression and patients’ demographics (Supplementary Table S3).

Five patients had two different variant types in a tumor specimen simultaneously (3 patients with V1 and V2 variants, 1 patient with V1 and V5′ variants, and 1 patient with V4 and V5′ variants); thus, a total of 64 variant types were detected in 59 patients. *EML4* accounted for the majority of *ALK*-fusion partners (96.9%). There were 2 patients with *KIF5B-ALK*. V1 and V3a/b were the most common variants. In the univariate analysis (Supplementary Table S3), the proportion of V3a/b was similar between the early and advanced stage but the V1 variant was more common in patients with metastatic disease (*P* = 0.035). Otherwise, no significant association between *ALK* variants and patients’ demographics was observed.

Interaction between PD-L1 expression and *ALK* variants was analyzed among 58 patients. There was no significant association between the PD-L1 status and the *ALK* variants (*P* = 0.925) (Fig. 2). The rate of PD-L1 expression in V1, V2, V3a/b, and other variants was 47.1%, 50.0%, 52.9%, and 62.5%, respectively.

### Impact of ALK variants and PD-L1 status on the outcome of ALK inhibitor(s) treatment

A total of 84 patients received *ALK* inhibitor(s) treatment while they were in advanced stage. The first-prescribed ALK inhibitor(s) were crizotinib in 48 patients and were second- or third-generation ALK inhibitor(s) in the remaining 36 patients (16 with alectinib, 12 with ceritinib, 6 with brigatinib, and 2 with lorlatinib). Among them, 43 patients received ALK inhibitor(s) as the first-line treatment. Thirty-five patients had brain metastasis at the start of ALK inhibitor(s) treatment and the baseline Eastern Cooperative Oncology Group performance status (ECOG PS) was 0–1 in 79 patients. The PD-L1 status and *ALK* variants were available in 69 and 43 patients, respectively. The progression-free survival (PFS) and overall survival (OS) of the entire population was 13.1 months (95% CI 10.4–15.7) and 53.6 months (95% CI NR-NR), respectively. Among the 73 patients with measurable disease, the objective response rate (ORR) and disease control rate (DCR) was 79.5% and 90.4%, respectively.

**Figure 3:**
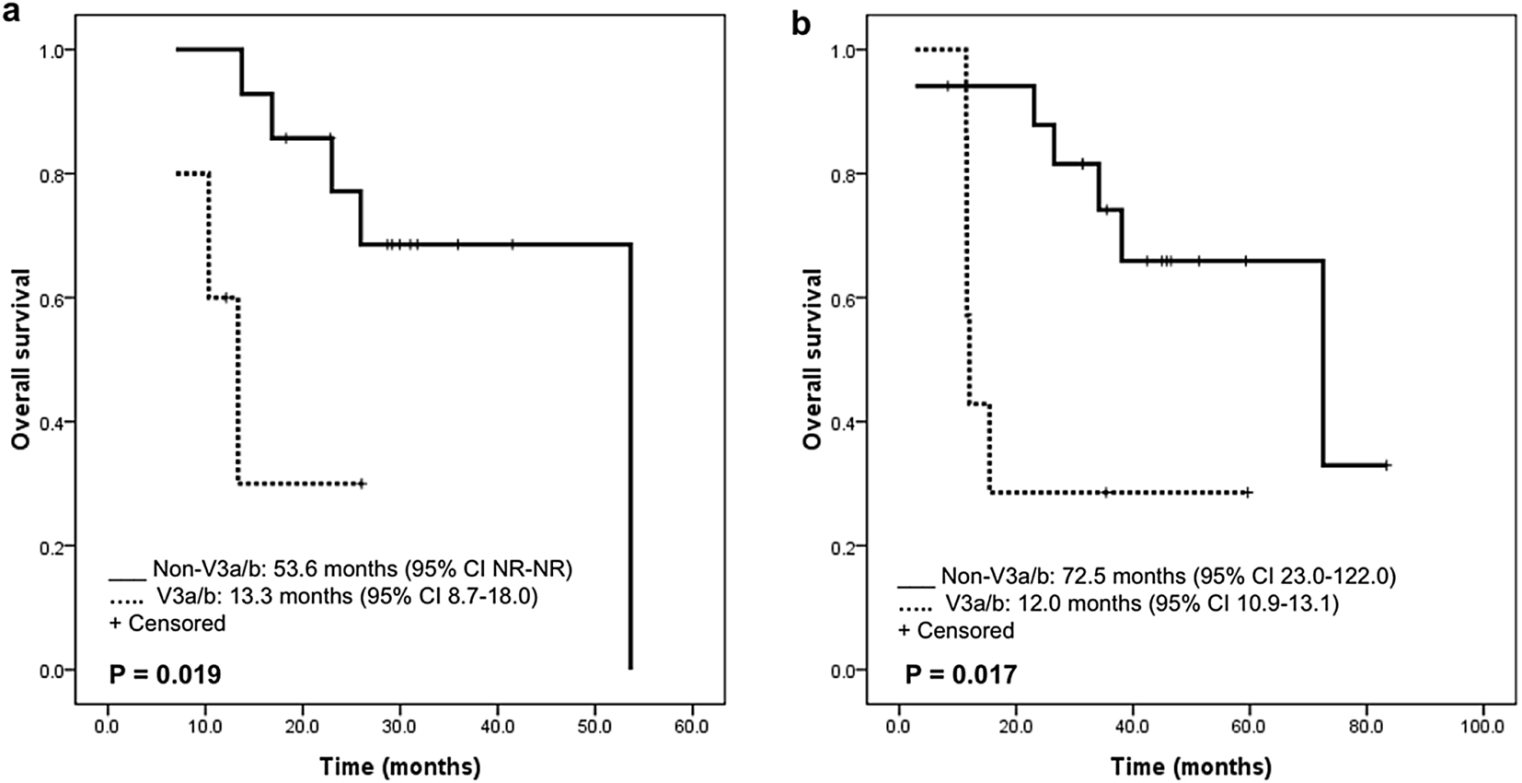
Impact of *ALK* variants on the overall survival of ALK inhibitor(s) (**a**) and platinum plus pemetrexed (**b**) treatment.

The results of univariate analysis are shown in Table 1. In the PFS analysis, ECOG PS 0–1, first-line use of ALK inhibitor(s), and the second- or third-generation ALK inhibitor(s) treatment were associated with a longer survival time (*P* = 0.002, 0.027, and 0.002, respectively). PD-L1 expression and *ALK* variant subtypes did not influence the PFS (*P* = 0.684 and 0.890, respectively). In the OS analysis, ECOG PS 0–1 and first-line use of ALK inhibitor(s) were associated with a longer OS (*P* < 0.001 and = 0.037, respectively). Patients received the second- or third-generation ALK inhibitor(s) also had a trend toward longer OS (*P* = 0.066). PD-L1 status did not influence the OS. Of note, patients with *ALK* V3a/b exhibited a worse OS (HR 4.24 [95% CI 1.01–17.79], *P* = 0.049) (Fig. 3a).

**Table 1:** Univariate analysis of progression-free survival and overall survival of patients receiving ALK inhibitor(s) (ALKi) (n = 84).

	PFS	*P* value^a^	OS	*P* value^a^
Age ≤ 50 versus > 50 years	1.48 (0.85–2.58)	0.171	0.55 (0.22–1.35)	0.192
Female versus male	0.84 (0.48–1.47)	0.540	1.02 (0.44–2.38)	0.960
NS versus C/FS	0.84 (0.48–1.50)	0.559	0.91 (0.38–2.17)	0.828
ECOG PS 0–1 versus 2–4	0.12 (0.03–0.45)	0.002	0.04 (0.01–0.17)	< 0.001
Brain metastasis no versus yes	0.41 (0.45–1.39)	0.790	0.76 (0.33–1.76)	0.516
ALKi 1st line or not	0.53 (0.30–0.93)	0.027	0.34 (0.13–0.94)	0.037
ALKi 2nd or 3rd-G versus 1st-G	0.36 (0.19–0.68)	0.002	0.36 (0.12–1.07)	0.066
PD-L1 positive versus negative^b^	0.88 (0.49–1.60)	0.684	0.65 (0.26–1.66)	0.370
ALK variants V3a/b versus others^c^	0.94 (0.39–2.27)	0.890	4.24 (1.01–17.79)	0.049

*PFS* progression-free survival, *OS* overall survival, *NS* non-smokers, *C/FS* current/former smokers, *ECOG PS* Eastern Cooperative Oncology Group Performance Status, *ALKi* ALK inhibitor(s).

^a^By logistic regression mode; presented by hazard ratio (95% CI).

^b^Available in 69 patients.

^c^Available in 43 patients.

In multivariate analysis, ECOG PS 0–1 was associated with both a longer PFS and OS (aHR 0.13 [95% CI 0.03–0.50], *P* = 0.003 and 0.06 [95% CI 0.01–0.28], *P* < 0.001, respectively). The second- or third-generation ALK inhibitor(s) treatment was associated with a longer PFS (aHR 0.42 [95% CI 0.21–0.83], *P* = 0.013) but not OS (*P* = 0.209). The first-line use of ALK inhibitor(s) was associated with a longer OS (aHR 0.30 [95% CI 0.10–0.92], *P* = 0.035), also. Of interest, the *ALK* variant 3a/b remained an independent predictor of a poor OS (aHR 5.10 [95% CI 1.22–21.25], *P* = 0.025).

**Figure 4:**
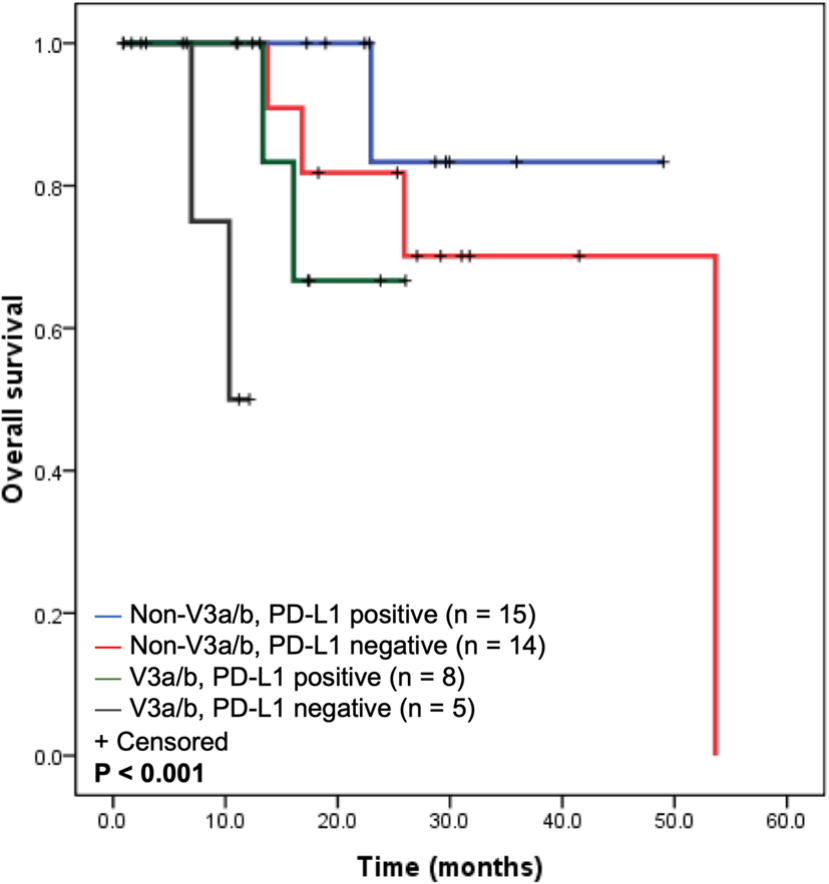
Impact of *ALK* variants and PD-L1 expression status on the overall survival of patients receiving ALK inhibitor(s).

While incorporating the PD-L1 status and *ALK* variants into the OS analysis (n = 42), the outcome of *ALK*-positive patients could be sorted by the two biomarkers. Patients with non-V3a/b/positive PD-L1 showed a trend towards longer OS, which was followed by non-V3a/b/negative PD-L1, V3a/b/positive PD-L1, and V3a/b/negative PD-L1 as shown in Fig. 4 (*P* < 0.001). Owing to the limited case numbers in each group, the *P* values of pairwise comparisons did not all reach the significant level (The *P* values of non-V3/PD-L1 positive vs. non-V3/PD-L1 negative, non-V3/PL-L1 positive vs. V3/PD-L1 positive, non-V3/PD-L1 positive vs. V3/PD-L1 negative, non-V3/PD-L1 negative vs. V3/PD-L1 positive, non-V3/PD-L1 negative vs. V3/PD-L1 negative, and V3/PD-L1 positive vs. V3/PD-L1 negative were 0.408, 0.194, 0.012, 0.551, 0.006, and 0.044, respectively).

### Impact of ALK variants and PD-L1 status on the outcome of chemotherapy

A total of 51 patients who had received platinum plus pemetrexed were included for outcome analysis. Among them, 14 patients had brain metastasis and the baseline ECOG PS was 0–1 in 47 patients. Forty-two patients received platinum plus pemetrexed as the first-line treatment. The platinum component was cisplatin in 45 patients. The PD-L1 status and *ALK* variants were available in 45 and 24 patients, respectively. The PFS and OS of the whole population was 9.6 months (95% CI 7.6–11.7) and 40.9 months (95% CI 13.4–68.4), respectively. Among the 47 patients with measurable disease, the ORR and DCR was 42.6% and 76.6%, respectively.

The results of univariate analysis are shown in Table 2. In PFS analysis, no factor was significantly associated with survival time. In OS analysis, there was a trend towards a longer OS in patients with ECOG PS 0–1 (*P* = 0.065). Of note, patients with *ALK* V3a/b had a worse OS (HR 4.24 [95% CI 1.18–15.21], *P* = 0.026) (Fig. 3b). In multivariate analysis, *ALK* variant 3a/b remained an independent predictor of a poor OS (aHR 9.62 [95% CI 1.90–48.80], *P* = 0.006).

**Table 2:** Univariate analysis of progression-free survival and overall survival of patients receiving chemotherapy (CT) with platinum plus pemetrexed (n = 51).

	PFS	*P* value^a^	OS	*P* value^a^
Age ≤ 50 versus > 50 years	0.80 (0.43–1.52)	0.502	0.71 (0.30–1.70)	0.443
Female versus male	1.87 (1.00–3.49)	0.051	1.76 (0.76–4.08)	0.190
NS versus C/FS	1.28 (0.69–2.39)	0.439	1.61 (0.65–3.96)	0.305
ECOG PS 0–1 versus 2–4	0.85 (0.26–2.80)	0.789	0.23 (0.05–1.10)	0.065
Brain metastasis no versus yes	1.69 (0.82–3.46)	0.153	1.73 (0.59–5.12)	0.322
CT 1st line or not	0.82 (0.34–1.97)	0.661	1.07 (0.32–3.63)	0.915
Cisplatin versus carboplatin	0.78 (0.48–1.27)	0.318	0.65 (0.37–1.12)	0.119
PD-L1: positive versus negative^b^	0.82 (0.42–1.61)	0.560	0.79 (0.30–2.07)	0.635
ALK variants V3a/b versus others^c^	1.44 (0.57–3.66)	0.445	4.24 (1.18–15.21)	0.026

*PFS* progression-free survival, *OS* overall survival, *NS* non-smokers, *C/FS* current/former smokers, *ECOG PS* Eastern Cooperative Oncology Group Performance Status, *CT* chemotherapy.

^a^By logistic regression model; presented by hazard ratio (95% CI).

^b^Available in 45 patients.

^c^Available in 24 patients.

While incorporating the PD-L1 status and *ALK* variants into the OS analysis (n = 24), patients with non-V3a/b/positive PD-L1 showed a trend towards longer OS, which was similar to that observed in the analysis of ALK inhibitor(s) treatment (Supplementary Fig. S1) (*P* = 0.086) (In pairwise comparisons, the *P* values of non-V3/PD-L1 positive vs. non-V3/PD-L1 negative, non-V3/PL-L1 positive vs. V3/PD-L1 positive, non-V3/PD-L1 positive vs. V3/PD-L1 negative, non-V3/PD-L1 negative vs. V3/PD-L1 positive, non-V3/PD-L1 negative vs. V3/PD-L1 negative, and V3/PD-L1 positive vs. V3/PD-L1 negative were 0.278, 0.107, 0.044, 0.256, 0.065, and 0.655, respectively).

## Discussion

It has now been well established that the treatment of advanced lung cancer is complex. The selection of treatment regimens is not solely based upon the histological types, but also the status of driver mutations and PD-L1 expression. Thus, accurate biomarker assessment plays a key role in personalized management. Currently, the PD-L1 status as well as at least four driver mutations, which include *EGFR*, *ALK*, *BRAF*, and *ROS1*, are recommended as the minimum necessary biomarker tests^16^. It may be even more complex to interpret the interaction between biomarkers. Herein, we report on the PD-L1 status and *ALK* variants of *ALK*-positive NSCLC patients. Moreover, we have analyzed their association and impact on the prognosis. The results suggest that the outcome of *ALK*-positive patients could be sorted by their *ALK* fusion subtypes and PD-L1 status.

In the present study, the *ALK* mutation rate among 1255 *EGFR*-wild type NSCLC patients was 9.9%, which was compatible with that of our previous report^17^. Within these patients, a younger age and smoking status were both independent predictors of *ALK* fusion. Herein, we further report the association with smoking dose. The higher smoking pack-years resulted in a lower *ALK* mutation rate. This phenomenon resembled that observed in the *EGFR* mutation^18^. Among the *EGFR*-wild type never/light smokers who were younger than 50 years of age, the *ALK* mutation rate could be as high as 23.0%.

There have been several studies investigating the association between *ALK* variants and patients’ outcome and the results of these studies were not consistent^10–12,19–26^. Since *ALK* fusion is a rare mutation in lung cancer, it would be worthwhile to explore the real-world conditions in different institutes. This would help to sketch a complete picture regarding the importance of *ALK* variants. Herein, we have summarized the results of prior studies and a total of 869 patients from twelve studies were analyzed (Supplementary Table S4). Similarly, the most common variants were V1 (38.2%) and V3a/b (33.4%). The only prospective study by Camidge et al. also suggested there was no significant impact of the *ALK* variants on PFS of first line crizotinib or alectinib treatment^10^. The overall survival had not yet matured. Regarding OS, five studies did not report their results, while four studies showed no significant difference. Additionally, Su et al. evaluated the OS of crizotinib treatment, while Christopoulos et al. evaluated the OS calculated from either the diagnosis of metastatic disease or from the initial diagnosis^19,25^. Both of the two studies suggested patients with the V3a/b variant having a worse outcome. In our study, we analyzed the OS of ALK inhibitor(s) and chemotherapy separately. The V3a/b variant remained a poor prognostic factor. Similar survival trends were observed in the subgroup analysis of crizotinib and second- or third-generation ALK inhibitor(s) treatment (Supplementary Fig. S2). Currently, we are only able to suggest that the V3a/b variant may predict a worse outcome. The real impact of *ALK* variants still requires further studies using a larger cohort.

There were five patients (8.5%) in our cohort with two *ALK* rearrangements. Of them, only one 49-year-old man received ALK inhibitor treatment. Although he achieved partial response to crizotinib, his PFS was only 6.7 months. In the study by Su et al.^25^, 22 of 110 patients harbored multiple *ALK* rearrangements and in turn were associated with a worse outcome. As compared with *EGFR* mutations, a previous study also suggested that complex mutations confer an inferior outcome to EGFR-TKI treatment^27^.

In the present study, we explored the PD-L1 status of *ALK*-positive NSCLC patients and analyzed its association with the *ALK* variants and patients’ outcome. The PD-L1 expression in NSCLC patients with driver mutation(s) may be diverse. Although *EGFR* mutation has been associated with a lower PD-L1 positive rate, patients with *ALK* or *ROS1* rearrangement have been reported to have a higher PD-L1 expression^28,29^. In our previous study, the PD-L1 positive and strong positive rate among *ALK*-positive patients were 46.7% and 13.3%, respectively^14^. Both of which were higher than that of the *EGFR*-mutant patients. Similarly, the PD-L1 positive and strong positive rate in the present study was 16.0% and 50.0%, respectively. These clinical observations could echo with prior studies which showed that EML4-ALK oncoprotein can upregulate the PD-L1 expression in lung cancer cells^30,31^. It is worth noting that there was no significant association between *ALK* variants and PD-L1 expression. It remains unclear whether different *ALK* rearrangements have a similar ability to induce PD-L1 expression and whether *ALK* fusion mutagenesis is independent from the PD1/PD-L1 checkpoint immune escape.

PD-L1 status may also have a diverse impact on the outcome of patients with driver mutations. Among patients harboring *EGFR* mutation, PD-L1 expression has been reported to be a poor prognostic factor^13,15^. By contrast, the status of PD-L1 expression solely did not influence the outcome of *ALK*-positive NSCLC patients. This scenario is similar with anaplastic large cell lymphoma, whose PD-L1 status is associated with *ALK* positivity but not its outcome^32^. Interestingly, the OS of *ALK*-positive NSCLC patients could be sorted while incorporating *ALK* variants and PD-L1 status together. In both ALK inhibitor(s) and chemotherapy treatment analysis, patients with non-V3a/b/positive PD-L1 showed a trend towards longer OS. Currently, the evidence is still limited for clinicians to apply immunotherapy to *ALK*-positive patients. Combined ALK inhibitor(s) and immunotherapy may lead to a higher toxicity^33^. In addition to prognosis estimation, biomarker assessment and patient sorting may provide an opportunity for subsequent research.

Recently, Yang et al. also reported the association of PD-L1 status with *ALK* variants and outcomes of ALK inhibitor(s) treatment^34^. The results suggested that *ALK* variant 3 and 5 (short variants) had higher PD-L1 expression rate and patients with positive PD-L1 was associated with worse outcome of crizotinib treatment. In this study, Yang et al. excluded patients with early disease and evaluated solely the efficacy of crizotinib treatment. Different patient population and study design may explain partly the dissimilar results with ours.

Owing to the rarity of this mutation and the limited case numbers in studies regarding with *ALK* fusion, we have to interpret the results with caution. However, more real-world data exploration may help to build up a consensus within the medical community. Further studies with larger cohorts are still required.

In conclusion, both a younger age and fewer smoking pack-years are associated with a higher *ALK* positive rate. The most common *ALK* variants are V1 and V3a/b. Although *ALK*-positive NSCLC patients possess a high PD-L1 expression rate, there is no significant association with *ALK* variant subtypes. The outcome of *ALK*-positive patients could be sorted by *ALK* variants and PD-L1 status.

## Methods

### Patients

To be eligible for the study, patients were required to have cytologically or pathologically confirmed NSCLC, known *ALK* status, clear demographic data, and survival follow-up. Patients were excluded if they had cancer with doubtful origin, another active malignancy, or *EGFR* mutation(s). This study was approved by the Institutional Review Board of Taichung Veterans General Hospital. Written informed consents for clinical data records, genetic and immunological testing were obtained from all patients. All methods were carried out in accordance with the approved guidelines and regulations.

### Data records and response evaluation

The lung cancer staging was done according to the 8th edition of the American Joint Committee on Cancer (AJCC) staging system^35^. Unidimensional measurements as defined by Response Evaluation Criteria in Solid Tumors (RECIST) version 1.1 were used in this study^36^. In whole study population, we accessed the *ALK* mutation rate in *EGFR*-wild type NSCLC patients and evaluated the association with clinical characteristics. In *ALK*-positive patients, we further analyzed the outcome of ALK inhibitor(s) and chemotherapy treatment, which emphasized the impact of PD-L1 status and *ALK* variants. The outcome of ALK inhibitor(s) treatment was focused on the first-prescribed ALK inhibitor, while the outcome of chemotherapy was focused on the platinum plus pemetrexed regimen.

### EGFR and ALK mutation detection

The *EGFR* mutations were detected by MALDI-TOF MS and the *ALK* mutation was detected by a fully automated IHC assay (Ventana IHC, Ventana, Tucson, AZ) using the pre-diluted Ventana anti-ALK (D5F3) Rabbit monoclonal primary antibody as previously described^17^.

### ALK variants testing

Total RNA extraction was performed using 40 μm-thick FFPE tumor specimens by the RecoverAll Total Nucleic Acid Isolation Kit (Thermo Fisher Scientific, USA) according to the users’ manual. The extracted RNA was quantified by the Qubit RNA HS Assay kit combined with a Qubit 2.0 fluorometer (Thermo Fisher Scientific, USA). Extracted RNA was reversely transcribed with the SuperScript VILO cDNA Synthesis Kit for further amplicon-based NGS experiments according to the manufacture’s instruction (ThermoFisher Scientific, USA).

The Ion Ampliseq Library kit 2.0 (Thermo Fisher Scientific, USA) with homemade customized multiplex primer pools was utilized for the library preparation. Primers specific for *ALK* fusions, including *EML4* and *KIF5B*, were designed in this study (Supplementary Table S1). The sequencing template and chip loading were carried out by the Ion 510 & Ion 520 & Ion 530 kit-chef and Ion S5 system sequencing kit with the Ion 510 chip kit or Ion 520 chip kit (Thermo Fisher Scientific, USA), separately. Raw sequencing data were processed using the Torrent Suite Software and aligned against the human genome (version hg19). To call a fusion, at least 20 reads of that specific fusion were required.

### PD-L1 status assessment

Formalin-fixed, paraffin embedded samples, whether they were tumor tissues or cell blocks from cytological specimens, were collected for PD-L1 IHC assay. All viable tumor cells on the slide prepared as 4-mm-thick and hematoxylin and eosin–stained sections were evaluated. The presence of at least 100 viable tumor cells was required for the specimen to be considered adequate for quantification of PD-L1 expression. PD-L1 status was accessed by the PD-L1 IHC 22C3 pharmDx as previously described^14^.

### Statistical methods

Univariate analyses of *ALK* mutation, *ALK* variants, and PD-L1 expression were performed using the Fisher’s exact test. The Kaplan–Meier method was used to estimate the survival time. Differences in survival time were analyzed by the log-rank test. The logistic regression model and Cox proportional hazard model were used for multivariate analyses of *ALK* mutation, *ALK* variants, PD-L1 expression, and survival outcomes^37^. All statistical tests were carried out using SPSS 15.0 (SPSS Inc., Chicago, IL, USA). Two-tailed tests and *P* values < 0.05 for significance were implemented.

## Supplementary information

Supplementary information.

## Data Availability

All data needed to evaluate the conclusions in the paper are present in the paper or the Supplementary Materials.
