# Supplementary material for: ALK variants, PD-L1 expression, and their association with outcomes in ALK-positive NSCLC patients
Source: Sci Rep. 2020 Dec 3;10:21063. doi: 10.1038/s41598-020-78152-1 (PMC7713243; doi:10.1038/s41598-020-78152-1)
Supplement: Supplementary file 1 — Supplementary information. [file 41598_2020_78152_MOESM1_ESM.pdf]

ALK variants, PD-L1 expression, and their association with outcomes in *ALK*-positive NSCLC patients

Gee-Chen Chang, Tsung-Ying Yang, Kun-Chieh Chen, Kuo-Hsuan Hsu, Yen-Hsiang Huang, Kang-Yi Su, Sung-Liang Yu, Jeng-Sen Tseng*

Supplementary Figure Captions

Supplementary Figure S1. Impact of *ALK* variants and PD-L1 expression on the overall survival of patients receiving platinum plus pemetrexed

Supplementary Figure S2. Impact of ALK variants on the overall survival of patients receiving Crizotinib (a) and second- or third-generation ALK inhibitor(s) (b)

Supplementary Figure S1

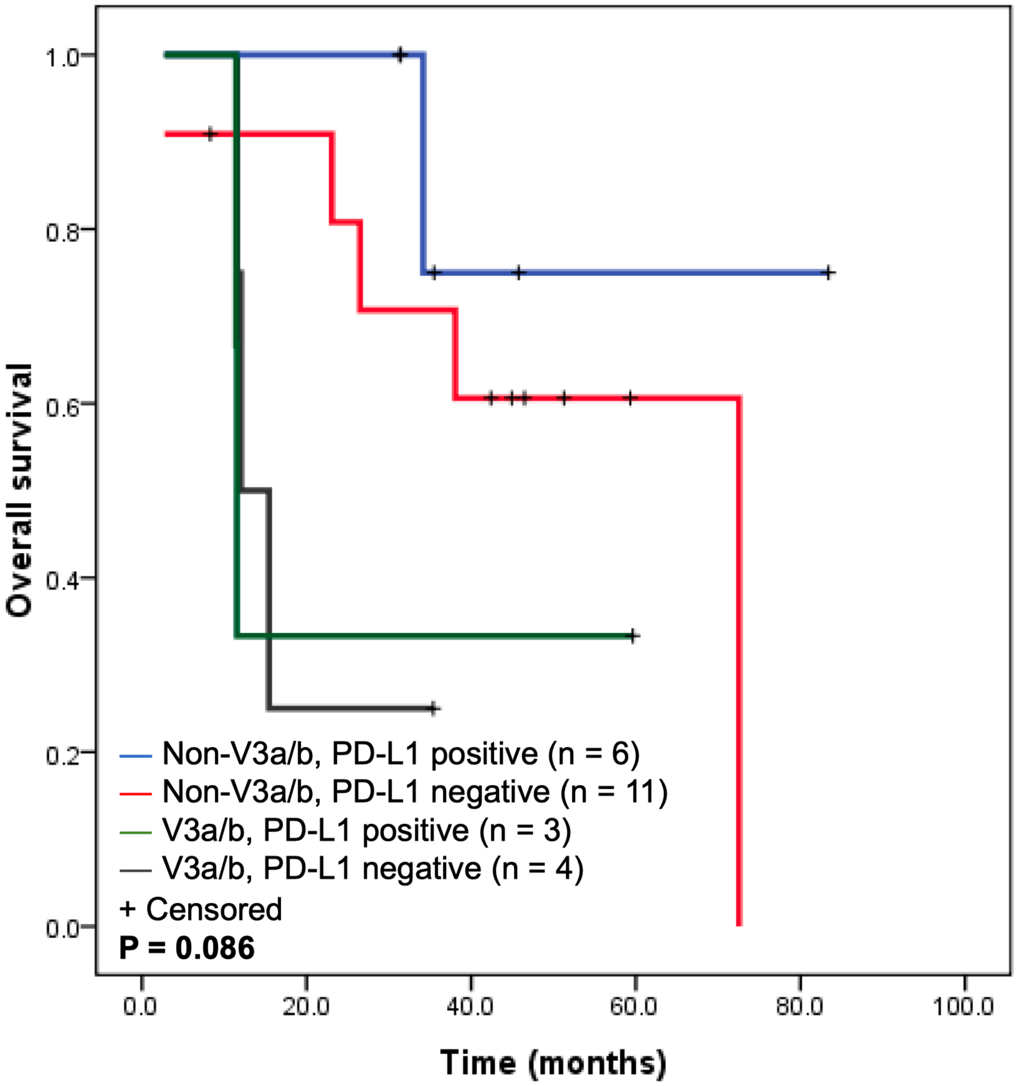

Supplementary Figure S2

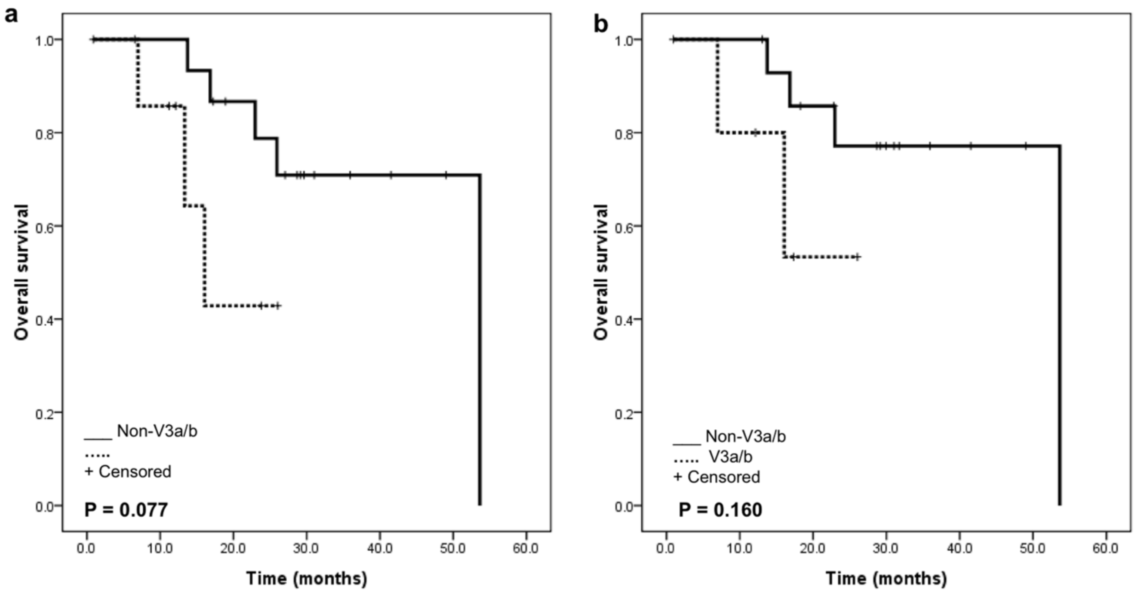

Supplementary Table S1. *ALK* variant subtypes detected by targeted next-generation sequencing system

**EML4**	**Non-EML4**
EML4 exon2 - ALK exon20	KIF5B exon15-ALK exon19
EML4 exon6 - ALK exon17	KIF5B exon15-ALK exon20
EML4 exon6 - ALK exon18	KIF5B exon17-ALK exon20
EML4 exon6 - ALK exon19	KIF5B exon24-ALK exon20
EML4 exon6 - ALK exon20	
EML4 exon7 - ALK exon20	
EML4 exon13-ALK exon20	
EML4 exon14-ALK exon20	
EML4 exon15-ALK exon20	
EML4 exon17-ALK exon20	
EML4 exon18-ALK exon20	
EML4 exon20-ALK exon20	

Supplementary Table S2. *ALK* mutation and demographic data

	ALK positive, n/N	(%)	P value[a]
Age			<0.001
≤ 50 years	53/259	(20.5)	
> 50 years	71/996	(7.1)	
Gender			0.005
Female	67/530	(12.5)	
Male	57/725	(7.9)	
Histology			0.034
Adenocarcinoma	119/1137	(10.5)	
Non-adenocarcinoma[b]	5/118	(4.2)	
Smoking status			0.004
Non-smokers	80/657	(12.2)	
Current/former smokers	44/598	(7.4)	

N, total cases; n, *ALK* positive patients.

[a]By the Fisher's exact test.

[b]Five *ALK* positive patients were 2 with squamous cell carcinoma, 2 with neuroendocrine carcinoma, and 1 with not-otherwise specified (NOS).

Supplementary Table S3. PD-L1 status, *ALK* variants, and patient characteristics

**PD-L1 status (n = 100)**				
	PD-L1 negative	PD-L1 low	PD-L1 strong	P value[a]
Age				0.962
≤ 50 years	22 (51.2)	14 (32.6)	7 (16.3)	
> 50 years	28 (49.1)	20 (35.1)	9 (15.8)	
Gender				0.611
Female	28 (50.9)	20 (36.4)	7 (12.7)	
Male	22 (48.9)	14 (31.1)	9 (20.0)	
Smoking				0.604
NS	33 (50.0)	24 (36.4)	9 (13.6)	
C/FS	17 (50.0)	10 (29.4)	7 (20.6)	
Histology				0.158
ADC	50 (51.5)	32 (33.0)	15 (15.5)	
Non-ADC	0 (0.0)	2 (66.7)	1 (33.3)	
Stage				0.810
I-III	15 (45.5)	12 (36.4)	6 (18.2)	
IV	35 (52.2)	22 (32.8)	10 (14.9)	
***ALK* variants (n = 59)**				
	V1	V3a/b	Others	P value[a]
Age				0.487
≤ 50 years	10 (33.3)	7 (23.3)	13 (43.3)	
> 50 years	7 (24.1)	11 (37.9)	11 (37.9)	
Gender				0.736
Female	10 (25.6)	12 (30.8)	17 (43.6)	
Male	7 (35.0)	6 (30.0)	7 (35.0)	
Smoking				0.468
NS	11 (24.4)	15 (33.3)	19 (42.2)	
C/FS	6 (42.9)	3 (21.4)	5 (35.7)	
Histology				N/A
ADC	17 (28.8)	18 (30.5)	24 (40.7)	
Non-ADC	0	0	0	
Stage				0.035
I-III	2 (10.0)	6 (30.0)	12 (60.0)	
IV	15 (38.5)	12 (30.8)	12 (30.8)	

NS, non-smokers; C/FS, current/former smokers; ADC, adenocarcinoma.

[a]By the Fisher's exact test.

Supplementary Table S4. Studies regarding with *ALK* variants and outcome of treatment

Author	Year Country	Design	No.[a]	Variants, % (V1/V2/V3/others)	Treatment	Timing of Tx.	PFS Difference	OS Difference
Yoshida[12]	2016 Japan	Retrospective	35	54.3/14.3/11.4.20.0	Crizotinib	Varied	V1 longer	N/A
Lei[20]	2016 China	Retrospective	61	36.1/11.5/29.5/23.0	Crizotinib	Varied	No	N/A
Cha[11]	2016 Korea	Retrospective	52	38.5/5.8/19.2/36.5	Crizotinib<br>Ceritinib<br>Platinum CT<br>PEM	Varied<br>Varied<br>Varied<br>Varied	No<br>No<br>No<br>V1 longer	N/A<br>N/A<br>N/A<br>N/A
Woo[26]	2017 Korea	Retrospective	54	33.3/11.1/44.4/11.1	Crizotinib<br>ALKi (1,2G)	Varied<br>Varied	V3 shorter<br>No	No<br>No
Li[21]	2018 China	Retrospective	60	23.3/15.0/33.3/28.3	Crizotinib	Varied	V2 longer	N/A
Christopoulos[19]	2018 Germany	Retrospective	67	38.8/10.4/50.7/0.0	ALKi (1,2G)<br>Platinum CT	Varied<br>Varied	V3 shorter<br>V3 shorter	V3 shorter
Lin[22]	2018 Mixed	Retrospective	129	42.6/6.2/39.5/11.6	Crizotinib<br>ALKi (2G)<br>Lorlatinib	1st ALKi<br>2nd ALKi<br>3rd ALKi	No<br>No<br>V3 longer	No
Mitiushkina[24]	2018 Russia	Retrospective	64	51.6/4.7/25.0/18.8	ALKi (1,2G)	Varied	No	No
Camidge[10]	2019 Global	Prospective	124	42.7/10.5/37.1/9.7	Alectinib<br>Crizotinib	First line<br>First line	No<br>No	N/A<br>N/A
Lin[23]	2019 Taiwan	Retrospective	54	42.6/11.1/33.3/13.0	Crizotinib	Varied	No	No
Su[25]	China	Retrospective	110[b]	29.1/7.5/30.6/32.8	Crizotinib	Varied	V3 or 5 shorter	V3 or 5 shorter
Chang (Current)	2020 Taiwan	Retrospective	59[b]	32.8/17.2/28.1/21.9	ALKi (1,2,3G)<br>Platinum + PEM	Varied<br>Varied	No<br>No	V3 shorter<br>V3 shorter
**Total**			**869**	**38.2/9.8/33.4/18.6**				

No., patient number; Tx., treatment; PFS, progression-free survival; OS, overall survival; N/A, not applicable; platinum CT, platinum-based doublet chemotherapy; PEM, pemetrexed; ALKi, ALK inhibitor(s); G, generation.

[a]Denote the patient numbers, of whom with *ALK* variant testing results.

[b]Some patients with two fusion variants in a tumor specimen simultaneously.
